# Forensic supportive housing programs: a scoping review

**DOI:** 10.3389/fpsyt.2026.1710135

**Published:** 2026-02-13

**Authors:** Kira Grachev, Nick Kerman, Alexander Ian Frederic Simpson, Artemis Igoumenou, Treena Wilkie, Vicky Stergiopoulos

**Affiliations:** 1 Centre for Addiction and Mental Health, Toronto, ON, Canada; 2 University Health Network, Toronto, ON, Canada; 3 Department of Psychiatry, University of Toronto, Toronto, ON, Canada

**Keywords:** forensic patients, forensic psychiatry, forensic supportive housing, scoping review, supportive housing

## Abstract

**Introduction:**

Forensic supportive housing (FSH) aims to support community reintegration and recovery of forensic psychiatric patients, while managing community risks. Although FSH is critical to the recovery process, the literature on FSH models and their outcomes is scant. A registered scoping review was undertaken to identify the models and outcomes of FSH internationally, and identify research gaps.

**Methods:**

Seven academic databases (Medline, Embase, PsycINFO, CINAHL Plus, Web of Science, Ebsco CINAHL, Ebsco Criminal Justice Abstracts, ProQuest) were searched, in addition to backward and forward citation searching. FSH models (program philosophy; support components and staffing composition) and their outcomes (e.g., re-hospitalization; re-offending; housing placement; well-being and quality of life) were examined.

**Results:**

A total of 4,084 articles were initially screened. Following full-text review of 82 articles, and citation searching, a total of 18 articles were included in the review. Most studies were descriptive. The program models identified varied in aims, program philosophy, and staffing models. Although most studies were uncontrolled, existing evidence suggests that FSH can support successful community tenures, and reduce risk of re-offending. Relationships with staff and fellow residents, along with opportunities to cultivate practical skills and self-efficacy, were identified as contributors to resident satisfaction in FSH. However, concerns of trans-institutionalization and limited resident autonomy and choice around housing options were identified in some settings, highlighting the need for greater attention to these programs.

**Conclusion:**

Research on FSH, an essential support for the successful community reintegration of forensic patients, is limited. Although existing literature highlights promising health, housing, and justice outcomes associated with FSH settings, more research is needed to establish best practices and support mental health recovery and safeguard the human rights of this population.

## Introduction

Over the past several decades, a series of key sociopolitical developments, including deinstitutionalization, legislative changes and the emergence of community-based mental health services, has shifted the care for people with serious mental illness from hospital to community settings (1, 2). Furthermore, the care and management of people with serious mental illness who engage in criminalized or socially disruptive behaviors has increasingly shifted to the jurisdiction of the criminal justice system, which has over time become a primary gateway to mental health services for this population (1, 2). An expanding number of patients are currently overseen by forensic rather than general psychiatric services (1). The steady growth of forensic populations is seen globally, including in Canada, where the number of individuals under forensic hospital supervision increased significantly – from 400 in 1992 to projections of 4,500 by 2015 (3). This increase has been identified as a significant concern, contributing to increased pressures on both forensic inpatient and outpatient services (4).

In Canada, individuals may be deemed Not Criminally Responsible (NCR) if, at the time of their offense, symptoms of a mental disorder prevented them from appreciating the nature or quality of their actions, or knowing their wrongfulness. NCR-accused individuals do not proceed through the conventional criminal justice pathway; instead, they fall under the authority of a provincial or territorial Review Board and enter the forensic mental health system (5). The Review Board issues one of three disposition orders: [1] detention, [2] conditional discharge, or [3] absolute discharge. Detention and conditional discharge dispositions encompass ongoing inpatient treatment or community-based support (5). In all cases, the Review Board balances public safety with the requirement to treat the NCR-accused individual in accordance with human rights legislation, selecting the necessary and appropriate “least onerous and least restrictive” option available (5).

Although these legal definitions and processes form the framework for the forensic mental health system in Canada, comparable frameworks exist across jurisdictions internationally. Furthermore, internationally, individuals in the forensic mental health system are met with a range of challenges that extend beyond the clinical severity of their conditions. In contrast to individuals criminally sentenced or those treated in the general psychiatric system, forensic patients often remain hospitalized for substantially longer periods – a pattern that is partially attributable to legal requirements and the limited availability of less restrictive alternatives (6).

It is worth noting that when community resources or required safeguards, such as supportive housing, are insufficient to satisfy disposition requirements, forensic patients may be required to remain in hospital longer, until suitable accommodations can be accessed (7). A U.S. study reported that forensic patients were institutionalized for periods ranging from two months to 20 years, based on data from four states (8). This reliance on high-acuity forensic hospital beds carries both financial and logistical burdens, as such beds are often scarce and costly (9). Furthermore, extended periods of institutionalization can make reintegration into the community particularly challenging for forensic patients, as they may face difficulties with performing daily activities of living, and managing societal norms and expectations (6). Upon eventual discharge, the dual stigma faced by NCR-accused individuals, including being labeled both “mentally ill” and “offender,” further complicates efforts at community reintegration (10). These labels can also limit access to community supports, including housing programs designed to promote stability and independence, thereby perpetuating cycles of institutionalization and isolation (1). As such, housing has a critically important role for this population, intersecting with fundamental issues of liberty and human rights, and potentially successful recovery.

Internationally, individuals under detention orders may transition into supervised community residences, whereas conditional discharge places individuals directly into the community under specified supports, often through forensic supportive housing (FSH) programs that typically provide case management, life skills training, and psychological support. Although FSH program structures and durations differ, most interventions offer 24-hour supervision and are focused on improving residents’ treatment adherence, practical living skills, and overall well-being (11, 12). Research findings suggest that FSH facilitates successful community reintegration (13), including lower rates of reoffending and psychiatric readmission compared with individuals discharged directly from custody to independent living (11). Moreover, participation in these programs has been associated with a greater likelihood of receiving an absolute discharge order (14).

Despite the recognized benefits and the serious ethical concerns surrounding prolonged involuntary detention, FSH is in short supply in many jurisdictions (9). Further, there are few principles or standards guiding program development, and a comprehensive review of the existing literature on forensic housing models and their outcomes has yet to be conducted. To support program and policy development and improvement efforts, a scoping review was conducted to examine FSH program models and outcomes. This approach was selected after a preliminary literature search confirmed a paucity of evidence in this area. The scoping review had two research questions: [1] What housing interventions for forensic patients leaving hospital have been described in the academic literature? and [2] What have been the outcomes associated with these forensic housing programs, including resident and other interest holder experiences?

## Methods

This scoping review was guided by the JBI Methodology for scoping reviews (15). The protocol was prospectively registered (https://osf.io/p8u62) (16). Review findings are reported in accordance with the Preferred Reporting Items for Systematic Reviews and Meta-Analyses extension for scoping reviews (PRISMA-ScR; see **Supplementary Table 1**) (17).

### Inclusion criteria

The inclusion criteria are outlined using the Population, Concept, and Context framework (15). The population of interest was adult forensic patients (≥18 years old). For the purpose of this review, a forensic patient was characterized as an individual with mental illness charged with, or convicted of, a criminal offence and who has obtained verdicts of Unfit to Stand Trial or NCR (and synonymous terms) on account of a mental disorder (5). For studies with mixed or general samples of non-forensic patients, ≥50% of participants were required to be forensic patients to be included in this review. The concept was defined as FSH programs that provided 24-hour supervision and onsite assistance to forensic patients. There were no limits placed on the outcomes examined in the reviewed literature. The context was FSH internationally, with equivalent terms being used to detect FSH and forensic patients considered across jurisdictions. Articles were considered eligible if they included one of more of the following: (a) program descriptions of FSH for forensic patients leaving hospital, (b) research outcomes or program evaluations of FSH for forensic patients, or (c) the experiences and perspectives of FSH interest holders (e.g., forensic patients, housing providers, clinicians).

Articles were also required to be published in a peer-reviewed academic journal between January 1^st^, 1990 and August 31^st^, 2024 (including advanced online publications), and written in either English or French. Exclusion criteria were conference abstracts, study protocols, editorials, books, book chapters, dissertations, and theses.

### Search strategy

A three-step search strategy was conducted by a research librarian to locate published studies. The first step was a keyword search of terms related to forensics and housing in Medline (Ovid) and PsycINFO (Ovid) to locate articles on the topic. This was followed by an analysis of text words used to describe the relevant articles in the title, abstract, and index terms fields. The second step was the development of a comprehensive search strategy in Medline (Ovid) using the identified keywords and index terms. The strategy was refined by the research team and adapted by the librarian for all searched databases. In the third step, reference lists of included articles were screened to identify additional relevant articles. A date limitation was included in the search strategy from 1990 to present. No further limits or filters were applied at the search level. Searches were conducted in September 2024, and the full search strategy for Medline (Ovid) is available in **Table 1**. One additional article was identified through citation searching.

**Table 1:** Medline search terms - ovid medline: epub ahead of print, in-process & other non-indexed citations, ovid medline® daily and ovid medline® <1946-present>.

#	Search query
1	exp Forensic Psychiatry/
2	Forensic Psychology/
3	Psychiatric Evaluation/
4	Forensic Assessment/
5	Competency to Stand Trial/
6	psychiatric jurisprudence/
7	Psychological Report/
8	Psychiatric Report/
9	mentally ill offenders/
10	criminally insane/
11	Insanity Defense/
12	McNaughton Rule/
13	(forensic* adj10 (assess* or client* or convict* or court* or criminal* or detain* or evaluat* or felon* or health* or hospital* or justic* or jurispruden* or law or legal* or offend* or outpatient* or patient$1 or mental* or psych*)).ab,hw,id,mf,ti.
14	("commitment of mentally ill" or "outpatient commitment" or "mentally ill commitments").ab,hw,id,mf,ti.
15	((psychiatric* or psychological*) adj1 jurisprudence).ab,hw,id,mf,ti.
16	((Psychological* or psychiatric*) adj1 Report).ab,hw,id,mf,ti.
17	(mental* adj3 (convict* or criminal* or detain* or felon* or offend*)).ab,hw,id,mf,ti.
18	criminally insane.ab,hw,id,mf,ti.
19	(conditional* adj3 (discharge or release$1)).ab,hw,id,mf,ti.
20	not criminally responsible.ab,hw,id,mf,ti.
21	("not guilty" adj5 (insanity or mental*)).ab,hw,id,mf,ti.
22	NGRI.ab,hw,id,mf,ti.
23	((insane or insanity) adj3 (acquit* or aquit* or criminal* or defense or defence or guilt*)).ab,hw,id,mf,ti.
24	M'Naghten*.ab,hw,id,mf,ti.
25	mcnaughton*.ab,hw,id,mf,ti.
26	(mental adj2 (competen* or incompeten*) adj10 (court$1 or criminal* or law*)).ab,hw,id,mf,ti.
27	(((unfit* or incompeten*) adj5 (trial* or tried)) and (court$1 or criminal* or law*)).ab,hw,id,mf,ti.
28	(secure adj1 (psychiatric or hospital or care)).ab,hw,id,mf,ti.
29	Housing/
30	residential facilities/
31	assisted living facilities/
32	group homes/
33	halfway houses/
34	Independent Living/
35	homeless person/
36	homelessness/
37	homeless/
38	housing insecurity/
39	housing instability/
40	exp Ill-Housed Persons/
41	((assisted or community or custodial or group or independent or justice-focused or reintegrat* or permanent or precarious or support* or temporary or transition*) adj2 (abode or accommodation or apartment$1 or cohabitation or dwelling or habitat or habitation or home$1 or homeless* or house$1 or housing or living or lodge or lodging or residence or rental or room or rooms or rooming or shelter or unhoused or un-housed)).ab,kf,kw,ti.
42	((abode or accommodation or apartment$1 or cohabitation or dwelling or habitat or habitation or home$1 or homeless* or house$1 or housing or living or lodge or lodging or residence or rental or room or rooms or rooming or shelter or unhoused or un-housed) adj2 (intervention* or model* or program* or service*)).ab,kf,kw,ti.
43	(abode or accommodation or apartment$1 or cohabitation or dwelling or habitat or habitation or home$1 or homeless* or house$1 or housing or living or lodge or lodging or residence or rental or room or rooms or rooming or shelter or unhoused or un-housed).ab,kf,kw,ti. /freq=2
44	or/1-28 [forensic/NCR]
45	or/29-43 [housing]
46	44 and 45 [all sets combined]
47	limit 46 to yr="1990 -Current"

### Information sources

The following seven databases were searched: [1] Ovid MEDLINE: Epub Ahead of Print, In-Process & Other Non-Indexed Citations, Ovid MEDLINE^®^ Daily and Ovid MEDLINE^®^ <1946-Present>, [2] APA PsycInfo <1806 to September 2024 Week 1>, [3] Embase Classic+Embase <1947 to 2024 September 10>, [4] Web of Science – Core Collection, [5] Ebsco CINAHL, [6] Ebsco Criminal Justice Abstracts, and [7] ProQuest Applied Social Sciences Index and Abstracts (ASSIA) (**Table 1**, **Supplementary Tables 4-9**).

### Evidence selection

**Figure 1:**
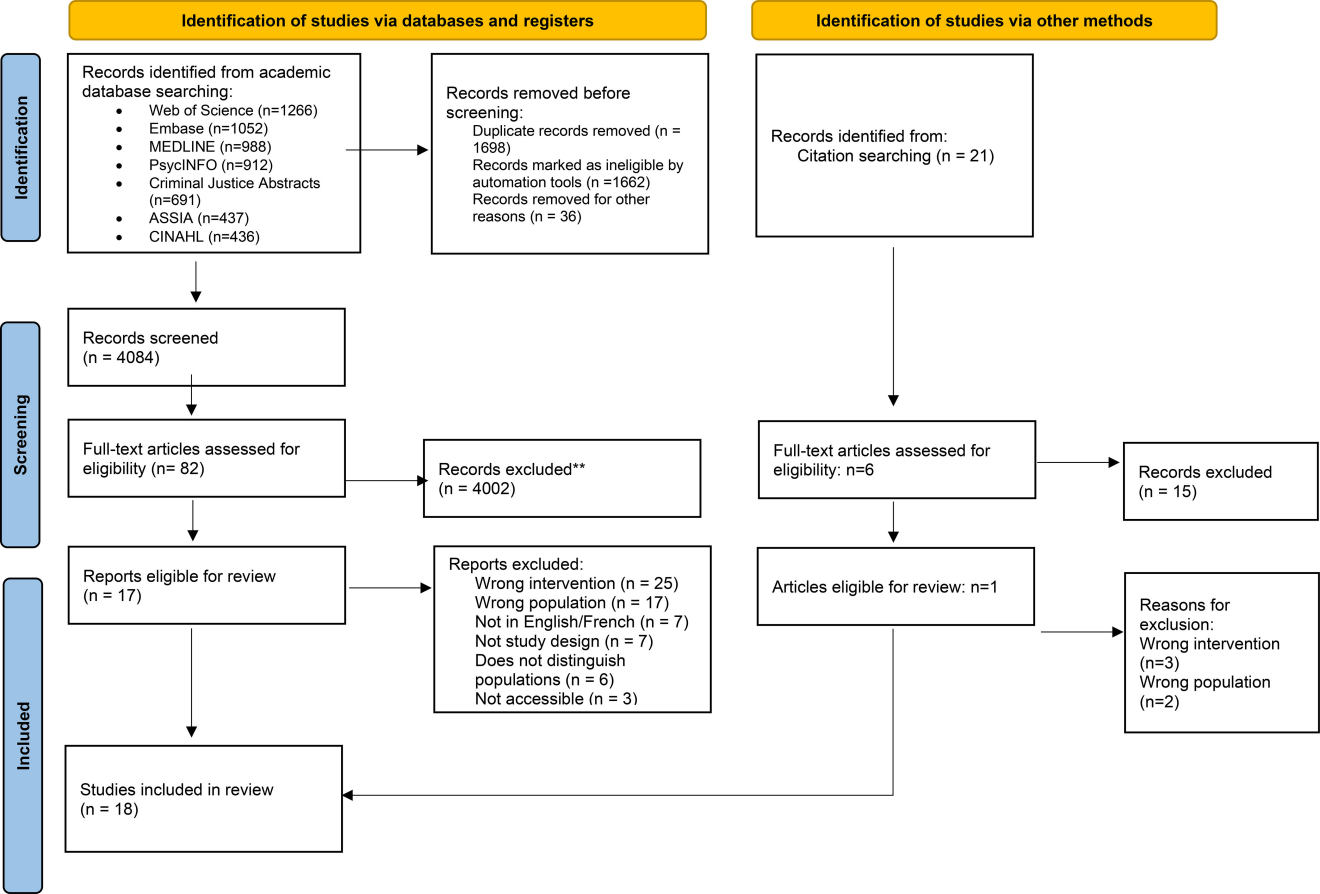
PRISMA 2020 flow diagram for new systematic reviews which included searches of databases, registers and other sources. Source: Page MJ, et al. BMJ 2021;372:n71. doi: 10.1136/bmj.n71.

Following the search, identified citations were imported into the software program Covidence and duplicates were removed. Titles and abstracts of the remaining citations were screened by two reviewers to determine their relevance to the review. A full-text review was conducted by two independent reviewers who met twice weekly to compare findings. Disputed articles (n=6) throughout the full text review process were resolved through consensus and, when necessary, consultation with senior team members. For articles requiring additional information to determine eligibility, the corresponding author was contacted to obtain the necessary details. The selection process and outcomes are shown in **Figure 1**.

### Data extraction and data synthesis

A data extraction template was developed to record the following items of the included articles: [1] title and author(s); [2] year of publication; [3] location of research/housing program; [4] study objectives and aims; [5] study design and method; [6] sample characteristics; [7] details on housing and support model (e.g. program philosophy, available supports); and [8] outcomes of interest. The data extraction template was piloted by two team members, who compared results from 3–5 articles and adjusted the template as necessary. Two members were then assigned articles and extracted information independently, periodically reviewing each other’s results. Extracted data were also periodically reviewed by a senior team member.

Critical appraisal of the included articles was conducted using a risk of bias assessment (**Supplementary Table 2**). Although optional in scoping reviews (17), this step was justified given the variability within the literature regarding study designs and program models, as well as the study’s objective to summarize the outcomes associated with these programs. Methodological quality was assessed using critical appraisal checklists from the British Medical Journal (BMJ) and the Joanna Briggs Institute (JBI), depending on study type (46, 47).

## Results

A total of 18 peer-reviewed journal articles were included in the review (**Figure 1**) after full text review of 82 articles and citation searching (11–14, 18–31). The included articles had a range of study designs, including program evaluation/descriptions (n=7), cross-sectional studies (n=3), cohort studies (n=3), qualitative studies (n=3), a case study (n=1), and a quasi-experiment (n=1). The geographic scope was narrow with articles originating from five countries, specifically the United Kingdom (U.K.) (n=6), Canada (n=5), the United States (U.S.) (n=4), Italy (n=2), and Ireland (n=1). The majority of the studies included in this review were published between 2013 and 2019 (n=12), whereas two articles were published in the 1990s and three articles were published within the 2000–2008 period (**Tables 2**, **3**, **Supplementary Table 3**).

**Table 2:** Description of included articles.

Author (Year)	Title	Country of study	Article objective	Article/Study design
Brown & Geelan (1998) (21)	Elliott House: Working with Mentally Disordered Offenders	United Kingdom	To describe the operation of Elliott House Probation and Bail Hostel.	Program description
Cherner et al. (2013) (13)	Findings of a Formative Evaluation of a Transitional Housing Program for Forensic Patients Discharged into the Community	Canada	To evaluate the implementation of a new transitional program in two cities against the planned program using a model-guided approach to evaluating program structures and processes.	Formative Program Evaluation
Cherner et al. (2014) (13)	Transitioning into the Community: Outcomes of a Pilot Housing Program for Forensic Patients	Canada	To describe client outcomes of the Transitional Rehabilitation Housing Pilot program in two cities.	Program evaluation
Chiringa et al. (2014) (14)	Reasons for recall following conditional discharge: explanations given by male patients suffering from dual diagnosis in a London Forensic Unit.	United Kingdom	To explore the perceived problems of conditionally discharged services users with a dual diagnosis that they attributed to their recall.	Qualitative Research
Clark et al. (2002) (22)	Psychiatric Probation Orders: Failed Provision or Future Panacea?	United Kingdom	To describe probationers of Elliott House, a specialist bail and probation hostel for mentally disordered offenders.	Cross-sectional study
Di Lorito et al. (2017) (19)	The Individual Experience of Aging Patients and the Current Service Provision in the Context of Italian Forensic Psychiatry: A Case Study	Italy	To explore the service experiences of a sample of patients aged 50 years and above living in one of the Italian Residence for the Execution of Security Measures (REMS).	Case Study
Geelan et al. (2000) (23)	A Bail and Probation Hostel for Mentally Disorder Defendants	United Kingdom	To report on the evaluation of the Elliott House probation hostel.	Mixed methods; program evaluation
Heard et al. (2019) (12)	Transitional Housing in Forensic Mental Health: Considering Consumer Lived Experience	Canada	To examine forensic mental health consumers' experiences in mental health and justice housing.	Qualitative study
Heilbrun et al. (1994) (24)	Community Placement for Insanity Acquittees: A Preliminary Study of Residential Programs and Person-Situation Fit	United States of America	To study the characteristics of community placements of insanity acquittees conditionally released following hospitalization and the interaction between individual characteristics of acquittees and their placements (referred to as "fit).	Program evaluation
Leadholm et al. (2018) (20)	High-support hostel care for mentally disordered offenders: a description of service	United Kingdom	To describe the patient group, the model of care, and outcomes of residents in a 24-h supported hostel for mentally disordered offenders leaving secure setting over 13 years.	Program evaluation
Melnick (2016) (25)	Passageway: A Novel Approach to Success of Conditional Release - Principles and Constructs of the Model Residential Program for the Forensic Mentally Ill Patient	United States of America	To describe and analyze the principles and constructs of Passageway, a model residential program for patients found not guilty by reason of insanity or those incompetent to proceed to Conditional Release.	Program description
Novosad et al. (2014) (27)	Statewide Survey of Living Arrangements for Conditionally Released Insanity Acquittees	United States of America	To define and review the current living situations of all insanity acquittees on conditional release in Oregon. To describe the current placement options in the state of Oregon on a single day.	Cross-sectional study
Novosad et al. (2016) (26)	Conditional Release Placements of Insanity Acquittees in Oregon: 2012-2014	United States of America	To describe demographic and system characteristics of insanity acquittees on conditional release; to describe specific living arrangements and movement between specific living arrangements while on conditional release.	Cohort study
Preti et al. (2008) (28)	A comparison between former forensic and non-forensic patients living in psychiatric residential facilities: A national survey in Italy	Italy	To compare the sociodemographic, clinical and treatment related characteristics of former forensic and non-forensic residents of psychiatric residential facilities.	Cross-sectional survey
Riordan et al. (2006) (30)	Possible Predictors of Outcome for Conditionally Discharged Patients - A Preliminary Study	United Kingdom	To identify variables among a cohort of conditionally discharged patients in the West Midlands that would predict whether an individual was more likely to be readmitted to hospital, involved in a serious incident, to be recalled to hospital or given an absolute discharge.	Retrospective cohort study
Salem et al. (2015) (11)	Supportive Housing and Forensic Patient Outcomes	Canada	To assess the influence of housing placements of forensic psychiatric patients conditionally discharged to the community on two main outcomes (i.e., recidivism and psychiatric readmissions).	Quasi-experimental study
Salem et al. (2016) (31)	Housing Trajectories of Forensic Psychiatric Patients	Canada	To describe the disposition and housing trajectories of individuals found Not Criminally Responsible on Account of Mental Disorder (NCRMD) in Quebec, and the factors that predict different trajectories.	Cohort study
Sweeney et al. (2013) (29)	Housing preferences of Irish Forensic Mental Health Service Users on Moving into the Community	Ireland	To identify and capture the views of mental health service users in a forensic setting on their housing preferences. To identify the strengths and weaknesses of the current housing services from the service users perspective.	Qualitative study

**Table 3:** Reported forensic supportive housing model components and their outcomes.

Authors (Year)	Supportive housing models & programs				Supportive housing outcomes			
	Program philosophy	Support components and staff mix	Transitional support	Substance use support	Re-hospitalization	Criminal justice	Housing placement	Wellbeing & housing satisfaction
Brown & Geelan (1998) (21)	X	X						
Cherner et al. (2013) (18)	X	X	X	X	X			X
Cherner et al. (2014) (13)		X	X		X	X		X
Chiringa et al. (2014) (14)		X			X			X
Clark et al. (2002) (22)	X	X			X		X	
DiLorito et al. (2017) (19)	X	X						X
Geelan et al. (2000) (23)	X	X				X		
Heard et al. (2019) (12)	X	X						X
Heilburn et al. (1994) (24)	X	X				X		X
Leadholm et al. (2018) (20)	X	X	X	X	X		X	
Melnick (2016) (25)	X	X	X	X		X		
Novosad et al. (2014) (27)	X	X						
Novosad et al. (2016) (26)	X	X				X	X	
Preti et al. (2008) (28)		X				X		
Riordan et al. (2006) (30)						X		
Salem et al. (2015) (11)	X				X	X		
Salem et al. (2016) (31)							X	
Sweeney & Shetty (2013) (29)			X					X

The critical appraisal revealed considerable variability in the methodological quality of the included studies (**Supplementary Table 2**). Some articles met several appraisal criteria, whereas a number of studies were assessed as low quality due to key limitations in methodology and reporting, such as unclear or inadequate description of data extraction methods and a lack of clarity regarding the instruments used to determine study outcomes. This variability in quality limits the ability to assess the findings with confidence and underscores the paucity of rigorous research in this important area.

### Resident characteristics

In all programs, the resident population was predominantly male, with four programs explicitly excluding women from eligibility (20–23). One study focused on examining the needs of older adults within the forensic system (19). Among studies reporting diagnostic characteristics, schizophrenia spectrum and related psychotic disorders were the most frequently identified conditions (11, 12, 20, 22, 23, 26, 28). Histories of substance use were also common; rates ranged from 43-82% as reported in five studies (20, 22, 23, 26, 30).

### Forensic supportive housing models and programs

#### Program philosophy

Twelve articles described the underlying program philosophy, discussing FSH models with considerable variation in objectives, provision of services, and staffing models. Generally, FSH programs employed rehabilitative and recovery-oriented principles, aimed at providing a pathway out of long-stay inpatient care through individualized support and community integration (13, 18–25). Among the 9 distinct FSH programs described in these studies, the number of residents ranged from 5–20 per program (13, 18–23, 26, 27). The majority of programs identified community reintegration and recovery as a primary objective, implementing approaches that included regularly assessing readiness for transition into community, promoting collaborative goal-setting, and facilitating the development of social networks (13, 18, 20). Two articles describing living arrangements of “insanity acquittees” in Oregon, U.S., discussed structured environments that, although offering similar staffing coverage and support services as other FSH programs, were characterized as more institutional in nature and described as “mini-state hospitals” (26, 27). In contrast, Italy’s *Residenze per l’Esecuzione delle Misure di Sicuezza* (REMS) facilities were described as explicitly promoting social inclusion, rather than containment, as the means to achieve recovery, distinguishing them from more custodial or institutional models of care (19). A few articles also highlighted the security measures implemented by FSH programs, which primarily involved some level of security, including controlled-entry and surveillance cameras (12, 19, 25, 27).

#### Support components and staffing compositions

Available supports in the described programs were extensive and commonly included case management, psychiatric and substance use support, financial management, and vocational and daily living skills training (12, 14, 18, 20, 22, 25–27, 29). Many programs offered a combination of onsite and off-site services (24–27). At Elliott House, a probation and bail hostel in the U.K., service continuity was encouraged by maintaining residents’ existing connections to off-site psychiatric and social services (23). However, logistical challenges hindered individuals’ ability to access services within their home communities, complicating access to psychiatric services during periods of acute crisis (23).

Programs adopted a range of staffing compositions. For example, at Elliott House, the staffing structure resembled that of most probation and bail hostels in the U.K., with roles including a senior probation officer and assistant wardens (21), whereas at REMS, the staffing was exclusively clinical personnel, with security staff only granted access during emergencies (19). There was no uniform approach to staffing intensity among the programs. REMS facilities, which were the only program to define staff-to-resident ratios, had 0.9 staff for 1 resident (19). Oregon’s Residential Treatment Homes (RTH), Residential Treatment Facilities (RTF), and Secure Residential Treatment Facilities (SRTF) differed in their capacity and service models (27). RTHs generally accommodate ≤5 residents, whereas RTFs and SRTFs support between 6–16 residents (27). Staffing levels also vary in these programs, with RTHs and RTFs typically staffed with one direct care staff per 8-hour shift, whereas SRTFs have two direct care staff and one nurse per shift (27). In RTHs and RTFs, direct care staff primarily assist with activities of daily living, whereas most mental health services are externally offered through outpatient or day treatment delivered by the residential provider or through community mental health services. In contrast, SRTFs offer onsite clinical services, which reflect the most intensive staffing model of the three facilities. The need for strong support structures was highlighted by findings that residents with forensic mental health histories often required more intensive staffing than psychiatric residents without forensic backgrounds (28).

There was significant variation in who operated the FSH programs. For example, REMS facilities were operated by Italy’s National Health Service as small, secure residential units (19), whereas the Tilt Hostel in the U.K. was operated through a partnership between a National Health Service provider and a charitable organization (20). By comparison, Passageway residences in the United States were established by a non-profit organization, with funding structured to promote both financial sustainability and resident accountability (25). These differences were also reflected in operational structures. Many FSH models underscored the importance of partnerships, collaborative care, and multidisciplinary teams to provide comprehensive support to residents (12, 13, 18, 20, 22). For example, Elliott House functioned through an interagency collaboration between forensic mental health and probation services, enabling regular meetings focused on risk assessment, individualized care planning, and coordination of discharge and aftercare processes (22). However, partnerships were not without challenges. Differences in treatment philosophies sometimes complicated interagency collaboration, as highlighted in a program evaluation of a Transitional Rehabilitation Housing Pilot (TRHP) in Ontario, which identified conflict between the hospital’s clinical model and a recovery-oriented community framework, as well as role ambiguity among staff across different sectors (18).

To monitor resident progress and maintain quality of care, most programs implemented routine processes among staff, such as regular case reviews, multidisciplinary team meetings, and ongoing risk assessments. Staff noted challenges in balancing supervision, as required by disposition orders, with recovery support (18). Weekly or monthly case reviews and team meetings were used to assess individual needs, revise support plans, and evaluate program effectiveness (20, 21). For example, the Tilt Hostel program used weekly clinical meetings with hostel and clinical team staff to assess the risk levels of each resident (20). Additionally, some programs placed emphasis on hiring and staff support as essential to maintaining quality care, with practices, such as team-building, reflective supervision, and values-based hiring, which reportedly led to staff retention and alignment with program goals (20, 25).

#### Supporting transitions from FSH to independent living

Structured, phased approaches were employed in programs to facilitate residents’ transitions from highly supported environments like FSH to independent living (13, 18, 20, 25). For instance, the Passageway program featured a seven-level system that gradually increased residents’ autonomy, starting with supervised residential care and progressing to strictly outreach services (25). Similarly, one TRHP program in Ontario, Canada, utilized a stepwise model with varying levels of support, beginning with a four-bedroom group residence and progressing to scattered-site apartments, which promoted autonomy alongside continued staff support (13, 18). Although several articles described successful implementation of phased transition models, one Irish study highlighted barriers to enacting such an approach. Participants in this study expressed support for step-wise reintegration, noting plans to transition to a lower-support hostel; however, this initiative was never realized due to funding and staffing resource limitations (29). Some programs also facilitated gradual acclimatization to the setting by encouraging prospective residents to visit multiple times prior to admission, helping to ease the adjustment process (20).

#### Supporting substance use health

Substance use policies and related supports within FSH were described in some of the articles. Some programs had strict abstinence requirements (25), whereas others made no mention of substance use expectations at all (19, 20, 28). For example, the Passageway program accepts individuals with a history of substance use problems, provided they demonstrate abstinence during their residency (25). During their stay, Passageway residents are required to attend 12-step meetings, with abstinence further supported through onsite services, such as supervised medication administration and substance use treatment (25). In two other programs, structured relapse prevention plans, including group and individualized treatments, were implemented for residents with a history of substance use (18, 20).

#### Forensic housing outcomes

The articles included in this review examined four outcome domains: [1] re-hospitalization; [2] criminal justice; [3] housing placement; [4] housing satisfaction and preferences (**Supplementary Table 3**).

#### Re-hospitalization

Six articles reported on re-hospitalization outcomes associated with transition to FSH and community living for forensic populations (11, 13, 14, 18, 20, 22). Re-hospitalization was framed as necessary and, at times, an appropriate response to challenges commonly experienced during community reintegration, including the management of mental health symptoms, substance use relapse, and medication non-adherence (13, 18, 22). Over half (55%) of residents in the TRHP program required at least one hospitalization over 18 months (13). Findings from another study highlighted the potential protective effect of FSH, with individuals residing in these programs experiencing lower rates of psychiatric readmission compared to those in independent housing (11). However, perceptions of FSH as protective against re-hospitalization were mixed. A qualitative study from the U.K. found that, among patients readmitted to a forensic unit from an FSH setting, poor or unclear communication with residence staff was commonly identified as a major factor contributing to their return to hospital (14). For example, one participant reported being re-hospitalized after missing an appointment due to receiving conflicting information from hostel staff, which was subsequently regarded as non-compliance by the supervisory authority (14).

#### Criminal justice

Eight articles examined criminal justice outcomes, such as changes in disposition orders and rates of recidivism. Overall, FSH was associated with positive trajectories in disposition orders and low rates of recidivism (11, 13, 24, 25).

Four articles discussed changes to disposition orders (23, 24, 26, 30). Although cancellation of disposition orders and return to hospital was reported in some studies, there was also evidence that FSH functioned as a protective factor against cancellations. Supportive and more structured settings were associated with lower rates of cancellation of orders of conditional release (26, 30). Moreover, one study reported that individuals on conditional discharge who resided in supportive housing were 4.5 times more likely to receive an absolute discharge compared to those living in other settings (30). One study developed a framework to assess both individual attributes of “insanity acquittees” and characteristics of FSH in which they lived (24). This framework was used to examine how the alignment between resident needs and program characteristics influenced outcomes (24). Preliminary findings suggested that poor fit – particularly treatment and monitoring – was associated with unsuccessful placements and an increased likelihood of cancellation of conditional discharge (24).

Low rates of recidivism were associated with FSH across studies (11, 13, 23, 28). In a quasi-experiment evaluating the role of housing type on criminal justice involvement among forensic patients, independent housing was associated with a significantly higher risk of reoffending compared to FSH (11). Furthermore, housing stability was found to be a stronger predictor of recidivism than traditional static risk factors (e.g., severity of index offense and length of detention) (11). Consistent with these trends, only four of the 83 individuals who lived at Elliott House over a 20-month period reoffended during their stay (23). Similarly, TRHP reported low rates of re-offending, with only 15% of residents committing a new offense over an 18-month period (13). One additional study concluded that individuals can be accommodated in FSH settings without posing substantial risks to public safety (28).

#### Housing placement

Four articles examined housing outcomes associated with FSH (20, 22, 26, 31). These studies found that severity of index offense was a major factor influencing access to FSH, with more severe offenses linked to reduced access (20, 26, 31). Additionally, a cohort study examining factors influencing the housing trajectories of forensic patients over a 36-month period found that clinical factors, including patients’ mental condition and prior hospitalizations, predicted housing trajectories for forensic patients (31). In particular, a higher number of hospitalizations prior to the index offense increased the likelihood of placement in supportive housing rather than independent housing (31). One study examined housing transitions following conditional release and found that individuals rarely moved between different levels of housing support after their placement. Individuals initially placed in less structured environments were likely to remain in such settings (26). While highly structured settings were found to provide greater stability in terms of reoffending, they were also associated with limited progression to less restrictive settings (26). Housing trajectories following transitions out of FSH were also examined in two studies. In one study, the majority of former residents moved into housing with fewer supports (20), whereas another study reported that most individuals were subsequently placed in alternative forms of supportive housing, with a smaller proportion (20%) achieving independent living (22).

#### Wellbeing, housing satisfaction, and preferences

Resident satisfaction and experiences with housing were discussed in seven articles (12–14, 18, 19, 24, 29). Overall, residents across various models of housing reported being satisfied with their experience in FSH, emphasizing the value of relationships with staff, the opportunity to develop practical skills, and the role of housing programs in fostering personal growth (12, 13, 18).

Residents highlighted the importance of interpersonal relationships in facilitating support and receiving care (12, 13, 18, 19). In a qualitative study examining residents’ lived experiences in Ontario, Canada, staff accessibility was seen as central to the development of therapeutic relationships, and shared experiences with peers and staff were described as enhancing quality of life by fostering a sense of belonging and contribution (12). However, not all interpersonal relationships were experienced positively; relational difficulties with both staff and peers were noted to have a detrimental impact on recovery, in some cases (13, 14). For example, in a qualitative study of a FSH program in the U.K., residents who had been recalled to hospital reported unclear and blurred interpersonal boundaries between residents and staff, and reported feelings of loneliness during their stays in FSH (14).

FSH and community living reportedly contributed to improvements in both functioning and quality of life (13, 18, 24, 29). For example, 83% of residents in one study affirmed at each follow-up interview that TRHP had positively contributed to their recovery (13). Despite this, residents’ evaluations of their recovery stayed relatively stable over time, with a slight decrease noted at the 18-month follow-up (13). Living in the community was also associated with reported improvements in both physical and mental health (13, 19). Residents identified various factors that enhanced their functioning and wellbeing, including independence, having privacy, building social connections among staff and community members, opportunities for personal growth, and gaining new skills and perspectives (12, 13, 18).

Residents described challenges with adjusting to a new environment in FSH, which was also an opportunity for personal growth (12, 13). These adjustments reportedly prompted the development of new coping strategies and life skills, which contributed to improved self-perception, including enhanced self-esteem and self-efficacy (12, 13). The opportunity to develop community living skills (e.g. budgeting, meal planning) within FSH settings, and to later apply them in more independent housing, was also identified as important for building confidence and resilience (12).

Self-determination and independence were perceived variably by residents; whereas some reported experiencing a sense of control, others indicated feeling a lack of agency in FSH settings (14, 19). Concerns were raised about the balance between supportive services and surveillance, with some residents perceiving the level of supervision as excessive, invasive, and controlling (14, 18). Further, residents in another study noted that a lack of freedom and uncertainty about the future had a particularly detrimental effect on their well-being (19).

Building on the theme of self-determination and control, residents also highlighted the significance of having a voice in decisions related to their housing (14, 29). Three articles noted that residents desired more transparency regarding housing options and hoped their preferences would be considered during the discharge process (14, 19, 29). For example, participants in one study indicated that their discharge conditions often determined their place of residence without regard for proximity to family, which was described as a barrier to accessing social support considered important to their recovery (14). Additionally, residents expressed a desire to transition from FSH to more independent living settings in some studies (14, 19, 29).

Generally, studies described FSH as meeting residents’ needs; however, the lack of meaningful and diverse activities contributed to feelings of social withdrawal and apathy among some residents in two studies (14, 19). A study examining aging residents in a REMS facility in Italy highlighted mixed views on age-specific activities and facilities, with generational differences influencing preferences for settings tailored to different age groups (19).

## Discussion

This scoping review examined the academic literature on FSH programs, with a focus on understanding the housing and support models, and their outcomes. Although there was considerable diversity in study methodologies and program structures, many FSH programs were grounded in recovery-oriented principles and supported community reintegration through individualized care plans and access to multidisciplinary teams (12, 13, 18–25). Residents generally reported positive experiences across several domains, including improvements in self-esteem, coping, and well-being, while rates of recidivism were low (11–13, 18, 23, 28). However, the review also identified challenges within some FSH settings related to the levels of supervision, lack of autonomy, and access to meaningful activities, which could hinder residents’ recovery experiences (14, 18, 19).

Operationally, programs offered a wide range of support services (e.g. case management, vocational training in some settings, and access to substance use treatment) (12, 14, 18, 20, 25–27). In terms of security, only a few articles described specific security features, mostly controlled-entry systems (12, 25, 27). In contrast, a small number of REMS programs in Italy left units unlocked and relied largely on clinical staff, reflecting greater emphasis on community integration (19). These variations in staffing and security reflect the diversity of program models and underlying philosophies. The primary objective across most programs was community reintegration and recovery rather than detention; however, some programs retained custodial features that even resembled “mini-state hospitals” (26, 27).

The programs included in this review, particularly those in the U.S. and the U.K., incorporated certain institutional features, such as more structured settings and a focus on risk management, though not all programs followed this approach. A comparative analysis of detention practices in forensic psychiatric care in three European countries highlighted that, in England and Wales, responsibility for patient recovery is largely the responsibility of clinicians, with a greater emphasis placed on managing perceived risks rather than on fostering patient autonomy (32). This is consistent with the findings of the present review. In contrast, the programs described in Canada and Italy were more oriented toward community reintegration and recovery, highlighting opportunities for cross jurisdictional learning to advance the field based on best practices.

Resident characteristics across included studies were largely homogeneous, with most residents being male and presenting with serious mental illnesses and histories of substance use, in alignment with previous research (1). Although many studies noted that substance use treatment was either integrated into housing programming or mandated as part of residence requirements, there were no programs that explicitly adopted a harm reduction approach and none of the studies examined how effectively existing programs addressed substance use-related outcomes. This gap is particularly concerning and warrants further investigation, as problematic substance use is a well-documented criminogenic risk factor, often serving as one of the most difficult barriers to achieving positive mental health and community reintegration (33).

FSH was associated with low rates of re-hospitalization and positive criminal justice outcomes. Although some studies observed that a proportion of residents experienced re-hospitalization, this was not regarded as a setback, but rather as part of the reintegration process, underscoring the complex and ongoing nature of recovery. FSH was also associated with positive disposition outcomes, such as absolute discharge, and low rates of recidivism (11, 13, 23, 28, 29). A disconnect was observed at times between the goals of community integration and the housing placements provided, and in some cases, it led to stagnation at a particular housing placement (26, 31). This theory-practice gap could be examined in future research, particularly given findings suggesting that residents in more structured housing experienced little movement to lower levels of care despite low recidivism rate, raising concerns about trans-institutionalization (26, 35).

Review findings showed that residents generally expressed satisfaction with their experiences in FSH, emphasizing the importance of relationships with staff and peers in fostering personal growth and recovery. This is consistent with previous research on supportive housing preferences among non-forensic individuals with serious mental illness, which has underscored the importance of reliable, ongoing support in fostering a sense of stability and psychological safety (36). Further highlighting the value of social connectedness, a study of adults with co-occurring disorders found that those in supervised housing were more likely to report a sense of community and comradery compared to those in independent settings (37). However, concerns were raised in some studies regarding a lack of autonomy, the level of surveillance, and the need for greater involvement in decision-making processes. These issues may be partially the result of little research on and attention to FSH programs compared to non-forensic supportive housing. For example, grounded in an extensive evidence base developed over the past several decades (38–45), supportive housing models for non-forensic individuals with serious mental illness have undergone considerable transformation, departing from custodial, risk management-focused approaches to embrace more recovery-oriented practices and philosophies, including Housing First (48). This research on non-forensic supportive housing has also revealed positive health and social outcomes, including reduced re-offending and resident satisfaction, with residents typically favoring models that support greater autonomy and choice (38–45, 48). As has been done with non-forensic supportive housing, future research on FSH programs should include rigorous evaluations of program effectiveness and use of implementation science tools to examine contextual barriers and facilitators associated with the development of FSH programs and their outcomes.

This review, bringing attention to FSH, an understudied area of enquiry, has several strengths, including rigorous methods, but also has some limitations. The studies included in this review were drawn from only a few countries, reflecting the limited focus that has characterized previous research on forensic populations (34). A further limitation of this scoping review is the relatively small number of articles identified, which were primarily descriptive. The variation in laws and terminology across different regional and international settings may have complicated the identification of relevant research, as well as the development of FSH program standards and comparative analysis. Finally, this focused review did not examine the literature on housing for justice involved individuals with mental illness outside of the forensic system, which might provide additional insights into models and practices to inform practice. This scoping review, however, highlights the paucity of research in this important area, and areas that need further attention, including the development of best practices in FSH settings and the opportunity for international research to advance the field. Future studies should include robust program descriptions, while future evaluations could consider longer follow-up durations, larger sample sizes, use of validated outcome measures, and more rigorous study designs.

## Conclusion

There is a dearth of research on FSH, an essential support for the successful community reintegration of forensic patients. Although existing literature highlights promising health, housing, and justice outcomes associated with FSH settings, more research is needed to establish best practices and support mental health recovery in this population.
