# Supplementary material for: Forensic supportive housing programs: a scoping review
Source: Front Psychiatry. 2026 Feb 13;17:1710135. doi: 10.3389/fpsyt.2026.1710135 (PMC12946025; doi:10.3389/fpsyt.2026.1710135)
Supplement: Supplementary file 1 [file SupplementaryFile1.docx]

**Supplemental Table 1.** Preferred Reporting Items for Systematic reviews and Meta-Analyses extension for Scoping Reviews (PRISMA-ScR) Checklist

| **SECTION** | **ITEM** | **PRISMA-ScR CHECKLIST ITEM** | **REPORTED ON PAGE #** |
| --- | --- | --- | --- |
| **TITLE** | | | |
| Title | 1 | Identify the report as a scoping review. | 1 |
| **ABSTRACT** | | | |
| Structured summary | 2 | Provide a structured summary that includes (as applicable): background, objectives, eligibility criteria, sources of evidence, charting methods, results, and conclusions that relate to the review questions and objectives. | 2 |
| **INTRODUCTION** | | | |
| Rationale | 3 | Describe the rationale for the review in the context of what is already known. Explain why the review questions/objectives lend themselves to a scoping review approach. | 3-5 |
| Objectives | 4 | Provide an explicit statement of the questions and objectives being addressed with reference to their key elements (e.g., population or participants, concepts, and context) or other relevant key elements used to conceptualize the review questions and/or objectives. | 5 |
| **METHODS** | | | |
| Protocol and registration | 5 | Indicate whether a review protocol exists; state if and where it can be accessed (e.g., a Web address); and if available, provide registration information, including the registration number. | 5 |
| Eligibility criteria | 6 | Specify characteristics of the sources of evidence used as eligibility criteria (e.g., years considered, language, and publication status), and provide a rationale. | 5-6 |
| Information sources* | 7 | Describe all information sources in the search (e.g., databases with dates of coverage and contact with authors to identify additional sources), as well as the date the most recent search was executed. | 7 |
| Search | 8 | Present the full electronic search strategy for at least 1 database, including any limits used, such that it could be repeated. | Supplemental Materials |
| Selection of sources of evidence† | 9 | State the process for selecting sources of evidence (i.e., screening and eligibility) included in the scoping review. | 6-7 |
| Data charting process‡ | 10 | Describe the methods of charting data from the included sources of evidence (e.g., calibrated forms or forms that have been tested by the team before their use, and whether data charting was done independently or in duplicate) and any processes for obtaining and confirming data from investigators. | 7 |
| Data items | 11 | List and define all variables for which data were sought and any assumptions and simplifications made. | 7 |
| Critical appraisal of individual sources of evidence§ | 12 | If done, provide a rationale for conducting a critical appraisal of included sources of evidence; describe the methods used and how this information was used in any data synthesis (if appropriate). | 7 |
| Synthesis of results | 13 | Describe the methods of handling and summarizing the data that were charted. | 7 |
| **RESULTS** | | | |
| Selection of sources of evidence | 14 | Give numbers of sources of evidence screened, assessed for eligibility, and included in the review, with reasons for exclusions at each stage, ideally using a flow diagram. | 7-8 & Figure 1 |
| Characteristics of sources of evidence | 15 | For each source of evidence, present characteristics for which data were charted and provide the citations. | 7-8 & Supplemental Materials |
| Critical appraisal within sources of evidence | 16 | If done, present data on critical appraisal of included sources of evidence (see item 12). | Supplemental Table 2 |
| Results of individual sources of evidence | 17 | For each included source of evidence, present the relevant data that were charted that relate to the review questions and objectives. | Table 3 & Supplemental Table 3 |
| Synthesis of results | 18 | Summarize and/or present the charting results as they relate to the review questions and objectives. | 7-15 |
| **DISCUSSION** | | | |
| Summary of evidence | 19 | Summarize the main results (including an overview of concepts, themes, and types of evidence available), link to the review questions and objectives, and consider the relevance to key groups. | 15-18 |
| Limitations | 20 | Discuss the limitations of the scoping review process. | 18 |
| Conclusions | 21 | Provide a general interpretation of the results with respect to the review questions and objectives, as well as potential implications and/or next steps. | 18 |
| **FUNDING** | | | |
| Funding | 22 | Describe sources of funding for the included sources of evidence, as well as sources of funding for the scoping review. Describe the role of the funders of the scoping review. | n/a |

**Supplementary Table 2.** Critical Appraisal Matrix

| **Article** | **Checklist** | **Item** | | | | | | | | | | | | | | | | | | |
| --- | --- | --- | --- | --- | --- | --- | --- | --- | --- | --- | --- | --- | --- | --- | --- | --- | --- | --- | --- | --- |
|  |  | **1** | **2** | **3** | **4** | **5** | **6** | **7** | **8** | **9** | **10** | **11** | **12** | **13** | **14** | **15** | **16** | **17** | **18** | **19** |
| Brown & Geelan (1998) | Not Applicable |  |  |  |  |  |  |  |  |  |  |  |  |  |  |  |  |  |  |  |
| Cherner et al. (2013) | Qualitative Research | Y | Y | Y | Y | Y | N | N | Y | U | Y |  |  |  |  |  |  |  |  |  |
| Cherner et al. (2014) | Quasi-Experimental | Y | Y | Y | N | Y | Y | Y | YN | NA | N |  |  |  |  |  |  |  |  |  |
|  | Qualitative Research | U | Y | Y | Y | Y | U | U | Y | Y | Y |  |  |  |  |  |  |  |  |  |
| Chiringa et al. (2014) | Qualitative Research | U | Y | Y | Y | Y | N | N | Y | Y | Y |  |  |  |  |  |  |  |  |  |
| Clark (2002 | Case Series | Y | N | U | Y | Y |  | Y | Y | Y | N | Y |  |  |  |  |  |  |  |  |
| DiLorito et al. (2017) | Cross-Sectional | Y | Y | Y | Y | U | Y | NA | Y | Y | NA | Y | NA | NA | Y | Y | Y | Y | U | Y |
| Geelan et al. (2000) | Cross-Sectional | Y | Y | N | Y | Y | Y | NA | Y | NA | Y | Y | U | NA | Y | Y | Y | U | U | U |
| Heard et al. (2019) | Qualitative  Research | Y | Y | Y | Y | Y | N | N | Y | Y | Y |  |  |  |  |  |  |  |  |  |
| Heilbrun et al. (1994) | Case Series | Y | Y | Y | U | Y | U | Y | N | Y | N | N |  |  |  |  |  |  |  |  |
| Leadholm et al. (2018) | Case Series | Y | Y | Y | U | U | U | Y | Y | Y | Y | U |  |  |  |  |  |  |  |  |
| Melnick (2016) | Not Applicable |  |  |  |  |  |  |  |  |  |  |  |  |  |  |  |  |  |  |  |
| Novosad et al. (2014) | Cross-Sectional | Y | Y | N | Y | Y | Y | NA | Y | NA | NA | Y | NA | NA | Y | Y | Y | U | U | U |
| Novosad et al. (2016) | Case Series | Y | Y | Y | Y | U | Y | Y | Y | Y | U |  |  |  |  |  |  |  |  |  |
| Preti et al. (2008) | Cross-Sectional | Y | Y | N | Y | Y | Y | U | Y | Y | Y | Y | N | NA | Y | Y | Y | Y | N | U |
| Riordan et al. (2006) | Case Series | Y | U | U | Y | Y | Y | N | N | N | Y |  |  |  |  |  |  |  |  |  |
| Salem et al. (2015) | Quasi-Experimental | Y | U | N | Y | N | N | Y | Y | Y |  |  |  |  |  |  |  |  |  |  |
| Salem et al. (2016) | Case Series | Y | Y | Y | Y | N | Y | Y | Y | N | Y |  |  |  |  |  |  |  |  |  |
| Sweeney & Shetty (2013) | Qualitative Research | Y | Y | Y | Y | Y | N | N | Y | Y | Y |  |  |  |  |  |  |  |  |  |

Y=Yes N=No U=Unclear NA=Not Applicable

Qualitative Research items: [1] Is there congruity between the stated philosophical perspective and the research methodology? [2] Is there congruity between the research methodology and the research question or objectives? [3] Is there congruity between the research methodology and the methods used to collect data? [4] Is there congruity between the research methodology and the representation and analysis of data? [5] Is there congruity between the research methodology and the interpretation of results? [6] Is there a statement locating the researcher culturally or theoretically? [7] Is the influence of the researcher on the research, and vice- versa, addressed? [8] Are participants, and their voices, adequately represented? [9] Is the research ethical according to current criteria or, for recent studies, and is there evidence of ethical approval by an appropriate body? [10] Do the conclusions drawn in the research report flow from the analysis, or interpretation, of the data?

Quasi-Experimental items: [1] Is it clear in the study what is the ‘cause’ and what is the ‘effect’ (i.e. there is no confusion about which variable comes first)? [2] Were the participants included in any comparisons similar? [3] Were the participants included in any comparisons receiving similar treatment/care, other than the exposure or intervention of interest? [4] Was there a control group? [5] Were there multiple measurements of the outcome both pre and post the intervention/exposure? [6] Was follow up complete and if not, were differences between groups in terms of their follow up adequately described and analyzed? [7] Were the outcomes of participants included in any comparisons measured in the same way? [8] Were outcomes measured in a reliable way? [9] Was appropriate statistical analysis used?

Cross Sectional items: [1] Were the aims/objectives of the study clear? [2] Was the study design appropriate for the stated aim(s)? [3] Was the sample size justified? [4] Was the target/reference population clearly defined? [5] Was the sample frame taken from an appropriate population base so that it closely represented the target/reference population under investigation? [6] Was the selection process likely to select subjects/participants that were representative of the target/reference population under investigation? [7] Were measures undertaken to address and categorize non-responders? [8] Were the risk factor and outcome variables measured appropriate to the aims of the study? [9] Were the risk factor and outcome variables measured correctly using instruments/measurements that had been trialed, piloted or published previously? [10] Is it clear what was used to determine statistical significance and/or precision estimates? [11] Were the basic data adequately described? [12] Does the response rate raise concerns about non-response bias? [13] If appropriate, was information about non-response described? [14] Were the results internally consistent? [15] Were the results presented for all the analyses described in the methods? [16] Were the authors’ discussions and conclusions justified by the results? [17] Were the limitations of the study discussed? [18] Were there any funding sources or conflicts of interest that may affect the authors’ interpretation of the results? [19] Was ethical approval or consent of participants attained?

Case Series items: [1] Were there clear criteria for inclusion in the case series? [2] Was the condition measured in a standard, reliable way for all participants included in the case series? [3] Were valid methods used for identification of the condition for all participants included in the case series? [4] Did the case series have consecutive inclusion of participants? [5] Did the case series have complete inclusion of participants? [6] Was there clear reporting of the demographics of the participants in the study? [7] Was there clear reporting of clinical information of the participants? [8] Were the outcomes or follow up results of cases clearly reported? [9] Was there clear reporting of the presenting site(s)/clinic(s) demographic information? [10] Was statistical analysis appropriate?

**Supplemental Table 3.** Description of Forensic Supportive Housing Programs and their Outcomes

| **Author (Year)** | **Description of Housing Program** | **Study Characteristics** | **Key Findings/ Outcomes** |
| --- | --- | --- | --- |
| Brown & Geelan (1998) | Elliott House is a specialist bail and probation hostel for mentally disordered offenders operated by West Midlands Probation Service and Reaside Clinic.    Staffing includes a medical team comprised of a consultant forensic psychiatrist, community psychiatric nurses and occupational therapists providing assessments, crises and stress management support and social skills training for certain residents; care plans are reviewed at weekly team meetings.    Efforts are made to re-establish and sustain connections with local psychiatric support services.    Philosophy of anti-racism and equality incorporated into operations, including culturally appropriate food and inquiries about ethnicity post-acceptance to hostel. | Program description | N/A |
| Cherner et al. (2013) | Two transitional housing programs (TRHP; City A, City B) cater to forensic mental health patients, providing 24-hour staff supervision.    On-site staff members include transitional support workers, case managers, nurses, and psychiatric professionals offering personalized assistance, including goal-setting, counselling and crisis intervention.    Residents have access to recovery-focused skills training (e.g. cooking, laundry) and social activities designed to enhance daily living abilities and to promote community integration. | Review of program documents at 2 sites (i.e. client charts, client recovery plans, minutes of project team meetings, project proposals), focus groups and interviews with 38 program staff, 23 key informants and 20 residents, and clinician ratings (e.g. Multnomah Community Ability Scale) | Program was implemented as planned, with referral processes volving consultation and information exchange among partners.    Intensive, individualized support and social and recreational activities were offered to residents, with fewer supports available in vocational and educational activities.    Program strengths included promotion of resident personal development and mental health recovery, opportunities to engage in “normal” community roles and reduced costs in comparison to inpatient hospital care. |
| Cherner et al. (2014) | Refer to Cherner et al. (2013) | Chart reviews, resident (N=20)  surveys at baseline, 6-, 12-, and 18- months, resident semi-structured interviews  and clinician ratings of mental health, functioning, substance use, etc. | Most residents experienced high functioning during their TRHP participation.    Residents reported increased independence, living arrangements with privacy, building social connections and learning new skills at TRHPs.    Majority of residents required re-hospitalization (55%), often due to medication non-adherence and substance use.    Reoffending was generally rare during TRHP participation. |
| Chiringa et al. (2014) |  | Qualitative semi-structured interviews with patients (N=6) readmitted to hospital following conditional discharge, review of patient records (demographic data, criminal offense, discharge conditions, recall) and field notes. | Most patients perceived that their recall to hospital was unjustified or not adequately understood and reported being treated as criminals and not patients during their readmission.  Poor treatment and care standards at mandated discharge hostels were considered by patients as a reason for recall, including a perceived lack of support, feelings of loneliness and boredom, and physical distance from familial supports. |
| Clark et al. (2002) | See  Brown & Geelan (1998) | Review of probation records and psychiatric case notes of 29 residents of Elliot House | Average age of residents during the study period was 26 years 4 months (range 17 years 4 months to 69 years 4 months); residents were all males, single, unemployed, and had histories of substance misuse. They committed a range of crimes and faced various sentences.    The average stay at Elliott House was 13.3 months; post-residence, 62.1% of residents moved to supported lodgings or hostels, and 20.7% went back to independent living; five had further complications, including psychiatric hospitalization or breached probation orders, with three returning to custody. |
| Di Lorito et al. (2017) | Residential units for the execution of security measures (REMS) are small residential units managed by Italy’s National Health Service.    REMS operate regionally, facilitating visitation from family and friends and supporting continuity of care upon discharge.      REMS have a maximum bed capacity of 20 beds; staff-to-patient ratio of 0.9-1; only clinical staff are on premises, with security personnel available only in case of a safety emergency. | Sociodemographic surveys, Camberwell Assessment of Needs Forensic-Short version, and semi-structured interviews with 5 residents 50 years or older, residing in the REMS for at least one year. | Number of identified needs among residents ranged from 14 to 21, with the greatest number of needs found for accommodation, food, daytime activities, physical health, information about their condition and treatment, psychological distress, basic education, access to telephone, money, benefits and treatment. The fewest number of needs was found for incontinence, sexual expression, and childcare.  Residents responded negatively when asked about overall happiness; forensic psychiatric setting was found to be negatively impacting on well-being, with residents describing lack of control over their lives and prospects for the future.    Residents expressed that facilities were not supportive enough of religious/spiritual needs and described barriers to sexual expression.    Residents expressed appreciation for the activities of everyday life including watching movies and reading, and for staff support.    Residents expressed a desire to be considered when addressing issues at the residence; issues described by the residents included inaccessible activities; lack of variety in the meals; and limited telephone use and length. |
| Geelan et al. (2000) | Elliott House accommodates up to 20 men and is staffed 24/7 by a senior probation officer, a probation officer, and assistant wardens, with at least two wardens on duty at all times.    The Reaside Clinic's multidisciplinary team conducts forensic psychiatric assessments and seeks to provide regular support to the hostel; the team is on call 24 hours a day for emergency support.    No formal risk assessment is conducted before resident acceptance.    The hostel has standard rules to other approved hostels in the UK, including a curfew from 11 pm to 6 am.    See also Brown & Geelan (1998) | A review of health and criminal records of 83 residents between August 1994 and April 1996. | Residents were all male; 45 of 83 residents were homeless prior to admission.    More than half (48 residents) had a history of alcohol use disorder and 39 had a history of psychosis; 32 residents had a history of deliberate self-harm; few had no prior convictions.    Common management challenges included ‘General Disturbance’ (50 cases), with varied behaviours such as loud singing and poor hygiene; only four residents reoffended while at Elliott House.    There was an increasing number of cases of residence conditions being breached (34 residents), often leading to custody; breaches were mostly due to absconding or rule violations.    A current charge for an acquisitive offense increased the risk of breaches; factors reducing the risk included being homeless, prior outpatient mental health care, and a charge for a sexual offense. |
| Heard et al. (2019) | Transitional housing operated by St. Leonard’s Community Services, London and Region (SLCS), in partnership with Southwest Centre for Forensic Mental Health Care.      SLCS facilities offer secure, highly structured and controlled entry residential settings with 24-hour staffing.    SLCS is committed to fostering “positive change” in forensic clients. Facilities utilize an “accountability-driven approach” to help individuals transition to more independent living in the community. | Qualitative interviews (n=6) with individuals residing in justice focused transitional housing operated by SLCS for a minimum of six months. | Residents described the importance of relationships with SLSC staff, including staff availability, as well as the provision of holistic support. The opportunity to share experiences with staff and peers, to contribute and to belong were described as enhancing quality of life for residents.    Residents valued that personal growth was enabled and described how residing at SLSC facilities enhanced personal agency including belief, awareness, confidence, esteem and hope.    The facility was reported to have enabled residents to gain practical skills, transferable to community living and participation. |
| Heilbrun et al. (1994) | Four Community Residential Placements in urban areas and five in smaller cities and towns in Florida, U.S.A.    Mental health services available on-site for three facilities; both on-site and off-site for three facilities; exclusively off-site for the remaining three facilities.    Employment was encouraged at four of the facilities; three facilities imposed restrictions on obtaining jobs (e.g. loss of disability payment). | Qualitative interviews with residents (N=13), and staff (from 9 residential facilities.    Interviews conducted every two months over a six-month period; fit ratings were determined by researchers across three dimensions: 1) monitoring, 2) treatment, 3) patient safety. | The "fit" between the resident and their placement was assessed across three domains: (1) degree and frequency of monitoring (2) scope and regularity of treatment  and (3) patient safety.    Three clients violated the conditions of their conditional release  within 6-months of their release; there was a suboptimal “fit” between resident and placement for two of the three individuals who violated the terms of their release.    Poor “fit” between resident and placement may play a role in re-hospitalization. |
| Leadhold et al., (2018) | Tilt Hostel is an 18-bed 24-hour supportive hostel, operated in partnership between Oxleas National Health Service (NHS) Trust and Bridge Support.    Tilt Hostel offers a pathway out of extended inpatient care. It provides services that support independent living, including managing finances and daily living activities related to tenancy.    Abstinence is a requirement, with care plans in place to manage risk of relapse which includes screenings, support groups and connection to local community services. | Review of Tilt Hostel referral and Medium Secure Unit referral databases between October 2023 and May 2016, including 81 residents. | Outcomes of 63 former Tilt Hostel residents included:    - Independent Tenancy: 22 residents (35%) successfully moved into independent housing.  - Less Supported Accommodation: 11 residents (17%) transitioned to less supported living.  - Other 24-Hour Hostel: 8 residents (13%) relocated to another 24-hour care facility.  - Readmission to Hospital: 19 residents (30%) were readmitted to hospital.  - Return to Prison: 2 residents (3%) returned to prison.  - Lost to Follow-Up: 1 resident could not be reached. |
| Melnick (2016) | The transitional residential program (Passageway) for forensic patients over 18 years found Not Guilty by Reason of Insanity (NGRI) or incompetent to proceed to trial for first-degree felonies and having an Axis I diagnosis. Program provides 24-hour supervised residential care, case management and individualized treatment planning, including psychiatric and healthcare services, group therapy, assistance with financial planning and vocational training.    Exclusion criteria include current substance use; previous substance use disorder and unwillingness to attend support group meetings regularly; suicidal or homicidal ideation; treatment refusal; program rule violations, or inability to function in a group environment. | Program description | Court mandated progressive privilege level system developed to support residents and the team responsible for their care.    The first four levels are designated as the residential phase and the final three levels are considered the outreach phase (in community); each level lasts approximately 3 months. |
| Novosad et al. (2014) | Structured settings (e.g. Residential Treatment Facility; Residential Treatment Home) delivering 24/7 care for a minimum of six residents each; staff support residents with daily living activities and skills training; mental health services are offered through outpatient or day programs provided on-site or at community mental health centres. | Secondary data from the Oregon Psychiatric Security Review Board (PSRB) on 389 insanity acquittees on monitored conditional release on a specific date February 1, 2014. | Structured settings make up 78% of all placements for individual on conditional release in Oregon. The rise in placements in residential treatment facilities and residential treatments homes compared to previous period suggests a shift toward housing more individuals in structured community environment. |
| Novosad et al. (2016) |  | Data from Oregon Psychiatric Security Review Board (PSRB) on 200 insanity acquittees placed on conditional release between January 1, 2012 and December 31, 2014.    Study compared first time conditional release from hospital (N=121) with not first conditional release from hospital (N=53) and court releases (n=26) | Most participants were middle-aged, single, Caucasian males; age range from 19 to 83 years; the majority of participants were diagnosed with a serious mental illness (87%), a history of substance abuse (82%), and prior interaction with mental health services (92%) and a history of prior arrests (87%); participants were charged with various offenses, ranging from murder to less severe felonies.    The most frequent placements were in the two most controlled settings (Secure Residential Treatment Facility and Residential Treatment Facilities/Homes, withother placements including adult foster homes and semi-independent living arrangements.    Severity of the criminal charge played a crucial role in the time to conditional release and the type of initial placement.    Placements in less structured environments had higher revocation rates.    Those placed in highly structured settings did not transition to less structured care levels. |
| Preti et al. (2008) | Psychiatric residential facilities in Italy with and without former forensic patients. | Comparison of residents with (N=193) and without (N=2,769) forensic mental hospital admission across 19.3% of Italian residential facilities (N=265).    Researchers assessed each facility and its residents, including  sociodemographic, clinical and treatment information; resident background information was collected by staff. | 64.7% of residential facilities housed 15% or fewer former forensic patients and 11.8% housed one-third or more former forensic patients. Facilities that served former forensic patients were typically larger (average of 15 beds vs 10 in other facilities) and employed a higher percentage of both specialized and general nurses to provide 24-hour staff coverage.    Former forensic patients received higher clinical severity ratings compared to non-forensic patients, were more likely to use antipsychotic medication and less likely to adhere to treatment plans. Former forensic patients exhibited a range of adaptive behaviours, including avoidance of at-risk behaviours and reduced tendency toward self-harm. |
| Riordan et al. (2014) |  | Chart reviews and administrative data files used to compare outcomes of patients conditionally discharged from a restricted hospital order into community in West Midlands between April 1, 1987 and April 1, 2000 (N=75) by housing type.    . | Patients were four and half times more likely to get an absolute discharge if they lived in supported accommodation than if they did not (OR=4.45, 95% CI 1.30, 15.32). |
| Salem et al. (2015) | Supportive housing, defined as housing with on-site mental health service support to promote activities of daily living, vocational and educational skills. | Review board files, health and criminal records were used to follow individuals found Not Criminally Responsible on account of Mental Disorder (NCRMD) conditionally discharged to supportive housing (n=223, 26.6%) vs. independent housing (n=614, 73.3%) in Quebec between May 2000 and April 2005; average length of follow-up was 743.86 days. | Risk of psychiatric readmission following conditional discharge was significantly related to housing type, when controlling for sociodemographic, clinical and criminological variables; independent housing put individuals at 1.36 times risk of psychiatric readmission when compared with supportive housing.    Risk of general recidivism and recidivism involving offenses against a person was reduced for individuals in supportive housing compared to those in independent housing. |
| Salem et al. (2016) | see Salem (205) | Disposition and housing status were coded for 936 individuals found NCRMD in Quebec  between May 2000 and April 2005 over a 36-month period. Sociodemographic, clinical, criminal history and risk assessments were extracted from Review Board files. | More hospitalizations prior to index offense were a predictor of supportive housing versus independent housing trajectory. A primary diagnosis of psychotic disorder did not influence the likelihood of placement in supportive housing. A primary diagnosis of a mood disorder was linked with less restrictive dispositions, decreasing the likelihood of belonging to the detention in hospital trajectory and supportive housing trajectories compared to the independent housing and absolute discharge trajectories    Individuals with higher index offense severity were significantly less likely to belong to supportive housing trajectory compared to detention. Clinical factors played a significant role in predicting less restrictive trajectories. |
| Sweeney et al. (2013) | Irish Forensic Mental Health Service consists of seven inpatient units, a hostel on hospital grounds, and a hostel away from hospital (24-hour staffed by nurses). | Qualitative interviews with nine male residents recruited among 31 eligible residents of a medium cluster unit and two hostels from the recovery and rehabilitation cluster. | Living alone was the preferred living choice among participants, allowing for maintenance of privacy.    Participants expressed desire for their housing preferences to be considered upon discharge; for social, medical and financial support; for accommodation similar to the community hostel; for phased reintegration into the community; and for information on housing options to facilitate informed decisions.    Existing legislation was considered a considerable obstacle to housing. |

Abbreviations:

NCR: Not Criminally Responsible

NCRMD: Not Criminally Responsible on account of Mental Disorder

NGRI: Not Guilty by Reason of Insanity

NHS: National Health Service

PSRB: Psychiatric Security Review Board

RTF/H: Residential Treatment Facilities/Homes

SRTF: Secure Residential Treatment Facility

**Supplementary Table 4. Search Strategy-APA PsycInfo <1806 to September 2024 Week 1>**

| **#** | **Search Query** |
| --- | --- |
| 1 | exp Forensic Psychiatry/ |
| 2 | Forensic Psychology/ |
| 3 | Psychiatric Evaluation/ |
| 4 | Forensic Assessment/ |
| 5 | Competency to Stand Trial/ |
| 6 | psychiatric jurisprudence/ |
| 7 | Psychological Report/ |
| 8 | McNaughton Rule/ |
| 9 | Psychiatric Report/ |
| 10 | mentally ill offenders/ |
| 11 | criminally insane/ |
| 12 | Insanity Defense/ |
| 13 | (forensic* adj10 (assess* or client* or convict* or court* or criminal* or detain* or evaluat* or felon* or health* or hospital* or justic* or jurispruden* or law or legal* or offend* or outpatient* or patient$1 or mental* or psych*)).ab,hw,id,mf,ti. |
| 14 | ("commitment of mentally ill" or "outpatient commitment" or "mentally ill commitments").ab,hw,id,mf,ti. |
| 15 | ((psychiatric* or psychological*) adj1 jurisprudence).ab,hw,id,mf,ti. |
| 16 | ((Psychological* or psychiatric*) adj1 Report).ab,hw,id,mf,ti. |
| 17 | (mental* adj3 (convict* or criminal* or detain* or felon* or offend*)).ab,hw,id,mf,ti. |
| 18 | criminally insane.ab,hw,id,mf,ti. |
| 19 | (conditional* adj3 (discharge or release$1)).ab,hw,id,mf,ti. |
| 20 | not criminally responsible.ab,hw,id,mf,ti. |
| 21 | ("not guilty" adj5 (insanity or mental*)).ab,hw,id,mf,ti. |
| 22 | NGRI.ab,hw,id,mf,ti. |
| 23 | ((insane or insanity) adj3 (acquit* or aquit* or criminal* or defense or defence or guilt*)).ab,hw,id,mf,ti. |
| 24 | M'Naghten*.ab,hw,id,mf,ti. |
| 25 | mcnaughton*.ab,hw,id,mf,ti. |
| 26 | (mental adj2 (competen* or incompeten*) adj10 (court$1 or criminal* or law*)).ab,hw,id,mf,ti. |
| 27 | (((unfit* or incompeten*) adj5 (trial* or tried)) and (court$1 or criminal* or law*)).ab,hw,id,mf,ti. |
| 28 | (secure adj1 (psychiatric or hospital or care)).ab,hw,id,mf,ti. |
| 29 | exp Housing/ |
| 30 | residential facilities/ |
| 31 | assisted living/ |
| 32 | assisted living facilities/ |
| 33 | group homes/ |
| 34 | halfway houses/ |
| 35 | Independent Living/ |
| 36 | homeless/ |
| 37 | homeless person/ |
| 38 | homeless mentally ill/ |
| 39 | housing insecurity/ |
| 40 | housing instability/ |
| 41 | ((assisted or community or custodial or group or independent or justice-focused or reintegrat* or permanent or precarious or support* or temporary or transition*) adj2 (abode or accommodation or apartment$1 or cohabitation or dwelling or habitat or habitation or home$1 or homeless* or house$1 or housing or living or lodge or lodging or residence or rental or room or rooms or rooming or shelter or unhoused or un-housed)).ab,hw,id,mf,ti. |
| 42 | ((abode or accommodation or apartment$1 or cohabitation or dwelling or habitat or habitation or home$1 or homeless* or house$1 or housing or living or lodge or lodging or residence or rental or room or rooms or rooming or shelter or unhoused or un-housed) adj2 (intervention* or model* or program* or service*)).ab,hw,id,mf,ti. |
| 43 | (abode or accommodation or apartment$1 or cohabitation or dwelling or habitat or habitation or home$1 or homeless* or house$1 or housing or living or lodge or lodging or residence or rental or room or rooms or rooming or shelter or unhoused or un-housed).ab,hw,id,mf,ti. /freq=2 |
| 44 | or/1-28 [forensic/NCR] |
| 45 | or/29-43 [housing] |
| 46 | 44 and 45 |
| 47 | limit 46 to yr="1990 -Current" |

**Supplementary Table 5. Search Strategy-Embase Classic+Embase <1947 to 2024 September 10>**

| **#** | **Search Query** |
| --- | --- |
| 1 | exp Forensic Psychiatry/ |
| 2 | Forensic Psychology/ |
| 3 | Psychiatric Evaluation/ |
| 4 | Forensic Assessment/ |
| 5 | Competency to Stand Trial/ |
| 6 | psychiatric jurisprudence/ |
| 7 | Psychological Report/ |
| 8 | Psychiatric Report/ |
| 9 | McNaughton Rule/ |
| 10 | mentally ill offenders/ |
| 11 | criminally insane/ |
| 12 | *Insanity Defense/ |
| 13 | (forensic* adj5 (assess* or client* or convict* or court* or criminal* or detain* or evaluat* or felon* or health* or hospital* or justic* or jurispruden* or law or legal* or offend* or outpatient* or patient$1 or mental* or psych*)).ab,hw,kf,kw,ti. |
| 14 | ("commitment of mentally ill" or "outpatient commitment" or "mentally ill commitments").ab,hw,kf,kw,ti. |
| 15 | ((psychiatric* or psychological*) adj1 jurisprudence).ab,hw,kf,kw,ti. |
| 16 | (mental* adj3 (convict* or criminal* or detain* or felon* or offend*)).ab,hw,kf,kw,ti. |
| 17 | (conditional* adj3 (discharge or release$1)).ab,hw,kf,kw,ti. |
| 18 | not criminally responsible.ab,hw,kf,kw,ti. |
| 19 | ("not guilty" adj5 (insanity or mental*)).ab,hw,kf,kw,ti. |
| 20 | NGRI.ab,hw,kf,kw,ti. |
| 21 | ((insane or insanity) adj3 (acquit* or aquit* or criminal* or defense or defence or guilt*)).ab,hw,kf,kw,ti. |
| 22 | M'Naghten*.ab,hw,kf,kw,ti. |
| 23 | mcnaughton*.ab,hw,kf,kw,ti. |
| 24 | (mental adj2 (competen* or incompeten*) adj10 (court$1 or criminal* or law*)).ab,hw,kf,kw,ti. |
| 25 | (((unfit* or incompeten*) adj5 (trial* or tried)) and (court$1 or criminal* or law*)).ab,hw,kf,kw,ti. |
| 26 | (secure adj1 (psychiatric or hospital or care)).ab,hw,kf,kw,ti. |
| 27 | Housing/ |
| 28 | residential facilities/ |
| 29 | assisted living/ |
| 30 | assisted living facilities/ |
| 31 | group homes/ |
| 32 | halfway houses/ |
| 33 | Independent Living/ |
| 34 | homeless/ |
| 35 | homeless person/ |
| 36 | homelessness/ |
| 37 | housing insecurity/ |
| 38 | Housing instability/ |
| 39 | exp Ill-Housed Persons/ |
| 40 | ((assisted or community or custodial or group or independent or justice-focused or reintegrat* or permanent or precarious or support* or temporary or transition*) adj2 (abode or accommodation or apartment$1 or cohabitation or dwelling or habitat or habitation or home$1 or homeless* or house$1 or housing or living or lodge or lodging or residence or rental or room or rooms or rooming or shelter or unhoused or un-housed)).ab,hw,kf,kw,ti. |
| 41 | ((abode or accommodation or apartment$1 or cohabitation or dwelling or habitat or habitation or home$1 or homeless* or house$1 or housing or living or lodge or lodging or residence or rental or room or rooms or rooming or shelter or unhoused or un-housed) adj2 (intervention* or model* or program* or service*)).ab,hw,kf,kw,ti. |
| 42 | ((abode or accommodation or apartment$1 or cohabitation or dwelling or habitat or habitation or home$1 or homeless* or house or housing or living or lodge or lodging or residence or rental or room or rooms or rooming or shelter or unhoused or un-housed) and (home or homes or house$1 or housing)).ab,hw,kf,kw,ti. |
| 43 | or/1-26 [NCR - forensics] |
| 44 | or/27-42 [housing] |
| 45 | 43 and 44 |
| 46 | limit 45 to yr="1990 -Current" |

**Supplementary Table 6. Search Strategy- EBSCO’s Criminal Justice Abstracts**

| **#** | **Search Query** |
| --- | --- |
| S23 | (S17 OR S18 OR S19) AND (S20 AND S21) |
| S22 | (S17 OR S18 OR S19) AND (S20 AND S21) |
| S21 | S17 OR S18 OR S19 |
| S20 | (S1 OR S2 OR S3 OR S4 OR S5 OR S6 OR S7 OR S8 OR S9 OR S10 OR S11 OR S12 OR S13 OR S14 OR S15 OR S16) |
| S19 | TI ( (abode or accommodation or apartment$1 or cohabitation or dwelling or habitat or habitation or home$1 or homeless* or house$1 or housing or living or lodge or lodging or residence or rental or room or rooms or rooming or shelter or unhoused or un-housed) ) OR AB ( (abode or accommodation or apartment$1 or cohabitation or dwelling or habitat or habitation or home$1 or homeless* or house$1 or housing or living or lodge or lodging or residence or rental or room or rooms or rooming or shelter or unhoused or un-housed) ) AND ( house* or housing* or home or homes ) |
| S18 | TI ( ((abode or accommodation or apartment$1 or cohabitation or dwelling or habitat or habitation or home$1 or homeless* or house$1 or housing or living or lodge or lodging or residence or rental or room or rooms or rooming or shelter or unhoused or un-housed) N2 (intervention* or model* or program* or service*)) ) OR AB ( ((abode or accommodation or apartment$1 or cohabitation or dwelling or habitat or habitation or home$1 or homeless* or house$1 or housing or living or lodge or lodging or residence or rental or room or rooms or rooming or shelter or unhoused or un-housed) N2 (intervention* or model* or program* or service*)) ) OR KW ( ((abode or accommodation or apartment$1 or cohabitation or dwelling or habitat or habitation or home$1 or homeless* or house$1 or housing or living or lodge or lodging or residence or rental or room or rooms or rooming or shelter or unhoused or un-housed) N2 (intervention* or model* or program* or service*)) ) |
| S17 | TI ( ((assisted or community or custodial or group or independent or justice-focused or reintegrat* or permanent or precarious or support* or temporary or transition*) N2 (abode or accommodation or apartment$1 or cohabitation or dwelling or habitat or habitation or home$1 or homeless* or house$1 or housing or living or lodge or lodging or residence or rental or room or rooms or rooming or shelter or unhoused or un-housed)) ) OR AB ( ((assisted or community or custodial or group or independent or justice-focused or reintegrat* or permanent or precarious or support* or temporary or transition*) N2 (abode or accommodation or apartment$1 or cohabitation or dwelling or habitat or habitation or home$1 or homeless* or house$1 or housing or living or lodge or lodging or residence or rental or room or rooms or rooming or shelter or unhoused or un-housed)) ) OR KW ( ((assisted or community or custodial or group or independent or justice-focused or reintegrat* or permanent or precarious or support* or temporary or transition*) N2 (abode or accommodation or apartment$1 or cohabitation or dwelling or habitat or habitation or home$1 or homeless* or house$1 or housing or living or lodge or lodging or residence or rental or room or rooms or rooming or shelter or unhoused or un-housed)) ) |
| S16 | (secure N1 (psychiatric or hospital or care)) |
| S15 | (((unfit* or incompeten*) N5 (trial* or tried)) and (court$1 or criminal* or law*)) |
| S14 | (mental N2 (competen* or incompeten*) N10 (court$1 or criminal* or law*)) |
| S13 | mcnaughton* |
| S12 | M'Naghten* |
| S11 | ((insane or insanity) N3 (acquit* or aquit* or criminal* or defense or defence or guilt*)) |
| S10 | ngri |
| S9 | ("not guilty" N5 (insanity or mental*)) |
| S8 | not criminally responsible |
| S7 | (conditional* N3 (discharge or release$1)) |
| S6 | criminally insane |
| S5 | (mental* N3 (convict* or criminal* or detain* or felon* or offend*)) |
| S4 | ((Psychological* or psychiatric*) N3 Report) |
| S3 | ((psychiatric* or psychological*) N3 jurisprudence) |
| S2 | "commitment of mentally ill" or "outpatient commitment" or "mentally ill commitments" |
| S1 | TI forensic* OR AB forensic* OR SO forensic* |

**Supplementary Table 7. Search Strategy-Applied Social Sciences Index & Abstracts (ASSIA)**

| **#** | **Search Query** |
| --- | --- |
| S1 | title(forensic* or "commitment of mentally ill" or "outpatient commitment" or "mentally ill commitments" or (mental* N3 (convict* or criminal* or detain* or felon* or offend*)) OR ((psychiatric* or psychological*) NEAR/3 jurisprudence) OR ((Psychological* or psychiatric*) NEAR/3 Report) OR (mental* NEAR/3 (convict* or criminal* or detain* or felon* or offend*)) OR criminally insane OR (conditional* NEAR/3 (discharge or release*)) OR "not criminally responsible" OR ("not guilty" NEAR/5 (insanity or mental*)) OR "ngri" OR ((insane or insanity) NEAR/3 (acquit* or aquit* or criminal* or defense or defence or guilt*)) OR M'Naghten* OR mcnaughton* OR (((unfit* or incompeten*) NEAR/5 (trial* or tried)) and (court$1 or criminal* or law*)) OR (secure NEAR/1 (psychiatric or hospital or care)) OR (mental NEAR/2 (competen* or incompeten*) AND (court$1 or criminal* or law*))) OR abstract(forensic* or "commitment of mentally ill" or "outpatient commitment" or "mentally ill commitments" or (mental* N3 (convict* or criminal* or detain* or felon* or offend*)) OR ((psychiatric* or psychological*) NEAR/3 jurisprudence) OR ((Psychological* or psychiatric*) NEAR/3 Report) OR (mental* NEAR/3 (convict* or criminal* or detain* or felon* or offend*)) OR criminally insane OR (conditional* NEAR/3 (discharge or release*)) OR "not criminally responsible" OR ("not guilty" NEAR/5 (insanity or mental*)) OR "ngri" OR ((insane or insanity) NEAR/3 (acquit* or aquit* or criminal* or defense or defence or guilt*)) OR M'Naghten* OR mcnaughton* OR (((unfit* or incompeten*) NEAR/5 (trial* or tried)) and (court$1 or criminal* or law*)) OR (secure NEAR/1 (psychiatric or hospital or care)) OR (mental NEAR/2 (competen* or incompeten*) AND (court$1 or criminal* or law*))) OR subject(forensic* or "commitment of mentally ill" or "outpatient commitment" or "mentally ill commitments" or (mental* N3 (convict* or criminal* or detain* or felon* or offend*)) OR ((psychiatric* or psychological*) NEAR/3 jurisprudence) OR ((Psychological* or psychiatric*) NEAR/3 Report) OR (mental* NEAR/3 (convict* or criminal* or detain* or felon* or offend*)) OR criminally insane OR (conditional* NEAR/3 (discharge or release*)) OR "not criminally responsible" OR ("not guilty" NEAR/5 (insanity or mental*)) OR "ngri" OR ((insane or insanity) NEAR/3 (acquit* or aquit* or criminal* or defense or defence or guilt*)) OR M'Naghten* OR mcnaughton* OR (((unfit* or incompeten*) NEAR/5 (trial* or tried)) and (court$1 or criminal* or law*)) OR (secure NEAR/1 (psychiatric or hospital or care)) OR (mental NEAR/2 (competen* or incompeten*) AND (court$1 or criminal* or law*))) AND summary(forensic* or "commitment of mentally ill" or "outpatient commitment" or "mentally ill commitments" or (mental* N3 (convict* or criminal* or detain* or felon* or offend*)) OR ((psychiatric* or psychological*) NEAR/3 jurisprudence) OR ((Psychological* or psychiatric*) NEAR/3 Report) OR (mental* NEAR/3 (convict* or criminal* or detain* or felon* or offend*)) OR criminally insane OR (conditional* NEAR/3 (discharge or release*)) OR "not criminally responsible" OR ("not guilty" NEAR/5 (insanity or mental*)) OR "ngri" OR ((insane or insanity) NEAR/3 (acquit* or aquit* or criminal* or defense or defence or guilt*)) OR M'Naghten* OR mcnaughton* OR (((unfit* or incompeten*) NEAR/5 (trial* or tried)) and (court$1 or criminal* or law*)) OR (secure NEAR/1 (psychiatric or hospital or care)) OR (mental NEAR/2 (competen* or incompeten*) AND (court$1 or criminal* or law*))) |
| S2 | title((assisted OR community OR custodial OR group OR independent OR justice-focused OR reintegrat* OR permanent OR precarious OR support* OR temporary OR transition*) NEAR/2 (abode OR accommodation OR apartment* OR cohabitation OR dwelling OR habitat OR habitation OR home* OR homeless* OR hous* OR housing OR living OR lodge OR lodging OR residence OR rental OR room OR rooms OR rooming OR shelter OR unhoused OR un-housed)) OR abstract((assisted or community or custodial or group or independent or justice-focused or reintegrat* or permanent or precarious or support* or temporary or transition*) NEAR/2 (abode or accommodation or apartment* or cohabitation or dwelling or habitat or habitation or home* or homeless* or hous* or housing or living or lodge or lodging or residence or rental or room or rooms or rooming or shelter or unhoused or un-housed)) |
| S3 | title((((abode OR accommodation OR apartment* OR cohabitation OR dwelling OR habitat OR habitation OR home* OR homeless* OR house* OR housing OR living OR lodge OR lodging OR residence OR rental OR room OR rooms OR rooming OR shelter OR unhoused OR un-housed) NEAR/2 (intervention* OR model* OR program* OR service*)))) OR abstract((((abode OR accommodation OR apartment* OR cohabitation OR dwelling OR habitat OR habitation OR home* OR homeless* OR house* OR housing OR living OR lodge OR lodging OR residence OR rental OR room OR rooms OR rooming OR shelter OR unhoused OR un-housed) NEAR/2 (intervention* OR model* OR program* OR service*)))) |
| S4 | title(( (abode or accommodation or apartment* or cohabitation or dwelling or habitat or habitation or home* or homeless* or house* or housing or living or lodge or lodging or residence or rental or room or rooms or rooming or shelter or unhoused or un-housed) ) OR ( (abode or accommodation or apartment* or cohabitation or dwelling or habitat or habitation or home* or homeless* or house* or housing or living or lodge or lodging or residence or rental or room or rooms or rooming or shelter or unhoused or un-housed) ) ) OR abstract(( (abode or accommodation or apartment* or cohabitation or dwelling or habitat or habitation or home* or homeless* or house* or housing or living or lodge or lodging or residence or rental or room or rooms or rooming or shelter or unhoused or un-housed) ) OR ( (abode or accommodation or apartment* or cohabitation or dwelling or habitat or habitation or home* or homeless* or house* or housing or living or lodge or lodging or residence or rental or room or rooms or rooming or shelter or unhoused or un-housed) ) ) AND ( house* or housing* or home or homes ) |
| S5 | [S2] OR [S3] OR [S4] |
| S6 | [S1] AND [S5] |

**Supplementary Table 8. Search Strategy-Web of Science – Core Collection**

| **#** | **Search Query** |
| --- | --- |
| 1 | ((((((((TS=(forensic*)) OR TS=(Competency to Stand Trial)) OR TS=( psychiatric jurisprudence))) OR TS=(McNaughton Rule)) OR TS=(mentally ill offenders)) OR TS=( criminally insane)) OR TS=(Insanity Defense)) |
| 2 | TS=((forensic* NEAR/5 (assess* or client* or convict* or court* or criminal* or detain* or evaluat* or felon* or health* or hospital* or justic* or jurispruden* or law or legal* or offend* or outpatient* or patient$1 or mental* or psych*)).) |
| 3 | TS=(("commitment of mentally ill" or "outpatient commitment" or "mentally ill commitments")) |
| 4 | TS=(((psychiatric* or psychological*) NEAR/1 jurisprudence)) |
| 5 | TS=((mental* NEAR/3 (convict* or criminal* or detain* or felon* or offend*))) |
| 6 | TS=(conditional* NEAR/3 (discharge or release$1)) |
| 7 | TS=("not criminally responsible") |
| 8 | TS=(NGRI) |
| 9 | TS=((insane or insanity) NEAR/3 (acquit* or aquit* or criminal* or defense or defence or guilt*)) |
| 10 | TS=(M'Naghten* OR mcnaughton*) |
| 11 | TS=((mental NEAR/2 (competen* or incompeten*) NEAR/10 (court$1 or criminal* or law*))) |
| 12 | TS=((((unfit* or incompeten*) NEAR/5 (trial* or tried)) and (court$1 or criminal* or law*))) |
| 13 | TS=((secure NEAR/1 (psychiatric or hospital or care))) |
| 14 | TI=housing OR AB=housing |
| 15 | TS=residential facilit* |
| 16 | TS=("group home" or "group homes") |
| 17 | TS=("halfway house" or "Halfway houses") |
| 18 | TS=("independent living") |
| 19 | (TI=((homeless or homelessness))) OR AB=((homeless or homelessness)) |
| 20 | TS=(ill-house*) |
| 21 | (TI=((assisted or community or custodial or group or independent or justice-focused or reintegrat* or permanent or precarious or support* or temporary or transition*) NEAR/2 (abode or accommodation or apartment$1 or cohabitation or dwelling or habitat or habitation or home$1 or homeless* or house$1 or housing or living or lodge or lodging or residence or rental or room or rooms or rooming or shelter or unhoused or un-housed))) AND AB=(((assisted or community or custodial or group or independent or justice-focused or reintegrat* or permanent or precarious or support* or temporary or transition*) NEAR/2 (abode or accommodation or apartment$1 or cohabitation or dwelling or habitat or habitation or home$1 or homeless* or house$1 or housing or living or lodge or lodging or residence or rental or room or rooms or rooming or shelter or unhoused or un-housed))) |
| 22 | (TI=(((abode or accommodation or apartment$1 or cohabitation or dwelling or habitat or habitation or home$1 or homeless* or house$1 or housing or living or lodge or lodging or residence or rental or room or rooms or rooming or shelter or unhoused or un-housed) NEAR/2 (intervention* or model* or program* or service*)))) AND AB=(((abode or accommodation or apartment$1 or cohabitation or dwelling or habitat or habitation or home$1 or homeless* or house$1 or housing or living or lodge or lodging or residence or rental or room or rooms or rooming or shelter or unhoused or un-housed) NEAR/2 (intervention* or model* or program* or service*))) |
| 23 | ((TI=(((abode or accommodation or apartment$1 or cohabitation or dwelling or habitat or habitation or home$1 or homeless* or house or housing or living or lodge or lodging or residence or rental or room or rooms or rooming or shelter or unhoused or un-housed) OR (home or homes or House$1 or housing)))) AND AB=((((abode or accommodation or apartment$1 or cohabitation or dwelling or habitat or habitation or home$1 or homeless* or house or housing or living or lodge or lodging or residence or rental or room or rooms or rooming or shelter or unhoused or un-housed) and (home or homes or House$1 or housing))))) |
| 24 | #1 OR #2 OR #3 OR #4 OR #5 OR #6 OR #7 OR #9 OR #8 OR #10 OR #12 OR #13 OR #11 |
| 25 | #14 OR #15 OR #16 OR #17 OR #18 OR #19 OR #20 OR #21 OR #22 OR #23 |
| 26 | #25 AND #24 |
| 27 | #26 Timespan: 1990-01-01 to 2024-09-06 |
| 28 | #26 and Article or Review Article (Document Types) Timespan: 1990-01-01 to 2024-09-06 |

**Supplementary Table 9. Search Strategy-EBSCO CINAHL**

| **#** | **Query** |  |
| --- | --- | --- |
| S23 | (S20 AND S21) AND (S20 AND S21) | **Limiters/Expanders** |
| S22 | (S20 AND S21) AND (S20 AND S21) | Limiters - Publication Date: 19900101-20251231 Expanders - Apply equivalent subjects Search modes - Proximity |
| S21 | (S17 OR S18 OR S19) | |
| S20 | S1 OR S2 OR S3 OR S4 OR S5 OR S6 OR S7 OR S8 OR S9 OR S10 OR S11 OR S12 OR S13 OR S14 OR S15 OR S16 | |
| S19 | TI ( (abode or accommodation or apartment$1 or cohabitation or dwelling or habitat or habitation or home$1 or homeless* or house$1 or housing or living or lodge or lodging or residence or rental or room or rooms or rooming or shelter or unhoused or un-housed) ) OR AB ( (abode or accommodation or apartment$1 or cohabitation or dwelling or habitat or habitation or home$1 or homeless* or house$1 or housing or living or lodge or lodging or residence or rental or room or rooms or rooming or shelter or unhoused or un-housed) ) AND ( house* or housing* or home or homes ) | |
| S18 | TI ( ((abode or accommodation or apartment$1 or cohabitation or dwelling or habitat or habitation or home$1 or homeless* or house$1 or housing or living or lodge or lodging or residence or rental or room or rooms or rooming or shelter or unhoused or un-housed) N2 (intervention* or model* or program* or service*)) ) OR AB ( ((abode or accommodation or apartment$1 or cohabitation or dwelling or habitat or habitation or home$1 or homeless* or house$1 or housing or living or lodge or lodging or residence or rental or room or rooms or rooming or shelter or unhoused or un-housed) N2 (intervention* or model* or program* or service*)) ) OR KW ( ((abode or accommodation or apartment$1 or cohabitation or dwelling or habitat or habitation or home$1 or homeless* or house$1 or housing or living or lodge or lodging or residence or rental or room or rooms or rooming or shelter or unhoused or un-housed) N2 (intervention* or model* or program* or service*)) ) | |
| S17 | TI ( ((assisted or community or custodial or group or independent or justice-focused or reintegrat* or permanent or precarious or support* or temporary or transition*) N2 (abode or accommodation or apartment$1 or cohabitation or dwelling or habitat or habitation or home$1 or homeless* or house$1 or housing or living or lodge or lodging or residence or rental or room or rooms or rooming or shelter or unhoused or un-housed)) ) OR AB ( ((assisted or community or custodial or group or independent or justice-focused or reintegrat* or permanent or precarious or support* or temporary or transition*) N2 (abode or accommodation or apartment$1 or cohabitation or dwelling or habitat or habitation or home$1 or homeless* or house$1 or housing or living or lodge or lodging or residence or rental or room or rooms or rooming or shelter or unhoused or un-housed)) ) OR KW ( ((assisted or community or custodial or group or independent or justice-focused or reintegrat* or permanent or precarious or support* or temporary or transition*) N2 (abode or accommodation or apartment$1 or cohabitation or dwelling or habitat or habitation or home$1 or homeless* or house$1 or housing or living or lodge or lodging or residence or rental or room or rooms or rooming or shelter or unhoused or un-housed)) ) | |
| S16 | (secure N1 (psychiatric or hospital or care)) | |
| S15 | (((unfit* or incompeten*) N5 (trial* or tried)) and (court$1 or criminal* or law*)) | |
| S14 | (mental N2 (competen* or incompeten*) N10 (court$1 or criminal* or law*)) | |
| S13 | mcnaughton* | |
| S12 | M'Naghten* | |
| S11 | ((insane or insanity) N3 (acquit* or aquit* or criminal* or defense or defence or guilt*)) | |
| S10 | ngri | |
| S9 | ("not guilty" N5 (insanity or mental*)) | |
| S8 | not criminally responsible | |
| S7 | (conditional* N3 (discharge or release$1)) | |
| S6 | criminally insane | |
| S5 | (mental* N3 (convict* or criminal* or detain* or felon* or offend*)) | |
| S4 | ((Psychological* or psychiatric*) N3 Report) | |
| S3 | ((psychiatric* or psychological*) N3 jurisprudence) | |
| S2 | "commitment of mentally ill" or "outpatient commitment" or "mentally ill commitments" | |
| S1 | TI forensic* OR AB forensic* OR SO forensic* | |
